# AGTS-Net: Anatomically Guided Two-Stage Network for Actionable Stroke Classification in Complete NCCT Studies

**DOI:** 10.3390/bioengineering13070827

**Published:** 2026-07-17

**Authors:** Rebeca Teruelo Diaz, Miguel Angel Vigil Berrocal, Iria Beltran Rodriguez, Vicente Rodriguez-Montequin

**Affiliations:** 1 Innvel Scientific Consulting, Calle Magnus Blikstad 58, 33207 Gijón, Spain; rebeca.teruelo@innvel.com; 2 Project Engineering Department, University of Oviedo, Calle Gonzalo Gutiérrez Quirós s/n, 33600 Mieres, Spain; montequi@uniovi.es; 3 Neurology Department, León University Hospital, Calle Altos de la Nava s/n, 24007 León, Spain; ibeltran@saludcastillayleon.es

**Keywords:** non-contrast computed tomography, stroke classification, anatomical pre-classification, region-specific convolutional neural networks, deep learning, explainable artificial intelligence, clinical decision support

## Abstract

Emergency stroke assessment using non-contrast computed tomography (NCCT) remains challenging because early ischemic signs may be subtle and anatomical variability across cranial scans can affect model performance. This study presents AGTS-Net (Anatomically Guided Two-Stage Network), an explainable deep learning framework designed to support stroke classification from complete NCCT studies. The framework was developed using a proprietary cohort of 99 patients, comprising 3440 NCCT slices annotated as hemorrhagic stroke, posterior ischemic stroke, anterior ischemic stroke, or no stroke. For each region-specific diagnostic classifier, data were split using the same scheme: 80% for training and 20% for testing, followed by a 20% validation split from the training subset. AGTS-Net first assigns each slice to a predefined anatomical region and then applies a region-specific classifier. Internal evaluation showed 0.99 anatomical-routing accuracy, and transfer learning experiments confirmed that anatomical regionalization improved classification over global models. A public Kaggle dataset of 7012 NCCT images was used only for external cross-domain evaluation of the anatomical pre-classifier, as compatible territorial diagnostic labels were unavailable. Grad-CAM maps visualized regions contributing to predictions. AGTS-Net is intended as a decision-support tool, providing actionable slice-level predictions and visual guidance during urgent stroke assessment.

## 1. Introduction

Stroke is a leading cause of adult mortality and disability worldwide [1]. It is clinically defined by the sudden onset of neurological deficits due to impaired cerebral blood flow, either from vascular occlusion (ischemic stroke) or vessel rupture (hemorrhagic stroke). Epidemiologically, ischemic strokes account for approximately 80% of cases, whereas hemorrhagic strokes represent about 20% [1,2]. Rapid and reliable differentiation between ischemia and hemorrhage is critical, as it directly determines immediate clinical decision-making [3,4].

Within ischemic stroke, lesions are commonly categorized by vascular territory into anterior (carotid) and posterior (vertebrobasilar) circulation. This distinction is clinically relevant, as it correlates with differences in presentation, prognosis, and therapeutic strategies [3].

In suspected stroke pathways, urgent imaging is required. Non-contrast cranial computed tomography (NCCT) is the first-line modality due to its availability and speed, particularly for excluding hemorrhage [5]. However, early detection in acute stages remains challenging due to acquisition-related limitations such as low contrast and limited resolution.

At early time points, intensity differences between healthy and affected tissue are often subtle and close to the noise level, which may result in apparently normal scans [6]. This limitation is exacerbated in anatomically complex regions such as the posterior fossa, where high-intensity structures (e.g., bone) coexist with low-contrast soft tissues, generating high-frequency artifacts that hinder the detection of subtle ischemic patterns [7]. Consequently, posterior circulation strokes are particularly prone to underdiagnosis or delayed detection. Given that up to 1.9 million neurons may be lost per minute without reperfusion, timely and accurate diagnosis is critical [8].

These challenges, together with observed diagnostic delays in real clinical settings (Hospital de León—[2]), motivate the development of robust automated methods.

Artificial intelligence (AI) methods have emerged as effective decision-support tools to reduce inter-observer variability and improve detection under weak-signal conditions, while accelerating clinical workflows [7]. From a machine learning perspective, this task involves several well-known challenges: weak signal, acquisition heterogeneity (e.g., resolution, reconstruction, windowing, and scanner configuration), and strong sensitivity to the processing pipeline, all of which can degrade model generalization [9,10].

**Figure 1:**
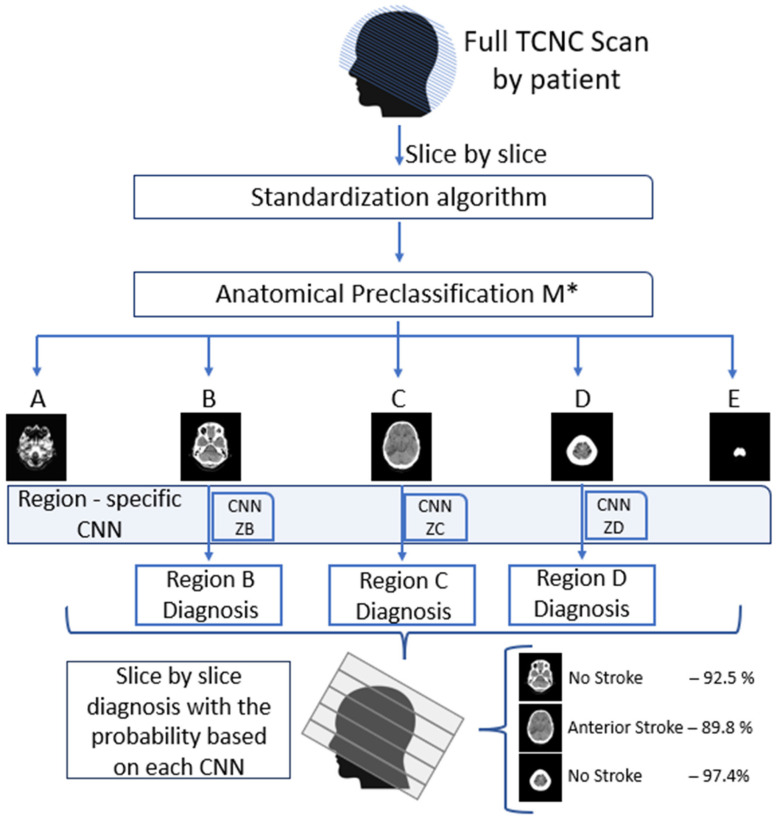
Schematic overview of the AGTS-Net cascaded system.

To address these limitations, we propose AGTS-Net (Anatomically Guided Two-Stage Network), a hierarchical cascaded framework designed to incorporate anatomical prior information into the classification pipeline. AGTS-Net formalizes a two-stage decision scheme in which the first stage performs anatomical region pre-classification across all NCCT slices into five regions (A, B, C, D and E). Conditioned on the assigned anatomical region, the second stage applies region-specific classifiers to perform four-class stroke classification: hemorrhagic stroke, anterior ischemic stroke, posterior ischemic stroke, and no stroke (Figure 1). In this work, the proposed cascade is evaluated using both a custom convolutional neural network (CNN) architecture and several transfer learning backbones, enabling a comprehensive assessment of the benefits of anatomical pre-classification across different deep learning models.

The main contributions are:

(i) Standardized preprocessing to reduce acquisition variability and improve model stability and transferability.
(ii) The validation of anatomical region pre-classification as an effective strategy for reducing task complexity and improving model robustness, particularly in challenging anatomical regions such as the posterior fossa.
(iii) Multi-class territorial classification (anterior/posterior) including a “no stroke” class, producing outputs aligned with real-world triage workflows.

To the best of our knowledge, this is the first approach for stroke detection from complete NCCT studies that jointly models’ presence, subtype, and vascular territory, closely reflecting emergency clinical workflows.

The remainder of this paper is organized as follows: Section 2 reviews related work; Section 3 details the proposed methodology; Section 4 describes the datasets; Section 5 presents experimental results; Section 6 discusses the limitations and future work; and Section 7 concludes the paper.

## 2. Related Work

Deep learning has become the dominant paradigm for image analysis due to its ability to learn hierarchical feature representations with minimal manual engineering. Among these approaches, CNNs are the de facto standard for image classification [11,12,13], as they exploit spatial dependencies through convolutional filtering and provide robustness to local transformations. These properties have enabled strong performance in medical imaging, where tasks are characterized by weak signal, high noise, and acquisition heterogeneity [14].

CNN-based methods have achieved competitive results in detection, classification, and segmentation tasks across medical imaging domains [15,16,17,18,19,20,21]. More recently, hybrid approaches combining CNNs with tailored training strategies, regularization, and architectural modifications have further improved robustness and performance [22,23,24]. Transfer learning is also widely adopted, particularly in small or heterogeneous datasets, by fine-tuning pretrained models to the target domain [16,25].

Despite these advances, generalization remains a key limitation. Model performance often degrades under domain shifts caused by variations in acquisition protocols, scanners, or clinical centers, highlighting the need for external validation [9]. In this context, rigorous standardization and explicit documentation of preprocessing pipelines are essential to ensure reproducibility and enable fair comparisons, as pipeline variability can significantly influence performance [26,27].

Within stroke imaging, existing approaches differ in both task formulation and data modality. They can be broadly categorized into:

(i) Binary detection (stroke vs. no stroke or ischemia vs. hemorrhage);
(ii) Basic multi-class classification (ischemia/hemorrhage/no stroke);
(iii) Territorial or subtype classification, often using modalities other than NCCT.

### 2.1. Binary Stroke Detection

Binary classification of stroke presence in NCCT typically achieves high internal performance but shows reduced robustness in external validation scenarios. For example, Abdi et al. [28] report 0.97 accuracy on internal data, decreasing below 0.90 on external datasets, indicating sensitivity to distribution shifts.

This behaviour is consistent with broader evidence. A systematic review and meta-analysis by Gete & Ayele [29] shows that, although overall accuracy is high, external validation consistently reduces performance. Sensitivity drops from 0.95 to 0.82, with additional declines in specificity and overall diagnostic accuracy.

These findings emphasize that robust deployment requires standardized preprocessing and explicit pipeline design. Moreover, binary detection is insufficient for clinical workflows, as differentiating ischemia from hemorrhage is a prerequisite for treatment selection.

### 2.2. Ischemia–Hemorrhage Classification

Classification between ischemic and hemorrhagic stroke has been extensively studied across imaging modalities [30]. High performance has been reported, particularly in Magnetic Resonance Imaging (MRI)-based studies, with accuracies above 0.94 [31] and up to 0.98 using CNNs [32], as well as near-perfect performance in hybrid approaches [24].

For NCCT, Gautam & Raman [33] report accuracy above 0.88 using CNN and transfer learning approaches. However, these studies are often limited by small datasets and lack of external validation, restricting generalizability.

Importantly, many of these approaches assume the presence of stroke and omit the “no stroke” class, which biases predictions toward false positives in real-world settings. Additionally, they do not explicitly address spatial localization or vascular territory.

### 2.3. Territorial Classification and Advanced Modalities

Territorial classification has been explored primarily for ischemic stroke using diffusion-weighted imaging (DWI), often combined with transfer learning approaches [34,35,36,37]. Some CNN-based methods also incorporate vascular territory classification and include a “no stroke” class [38,39].

Related tasks, such as intracranial hemorrhage detection in NCCT, have shown that deep learning can achieve reliable detection and localization, although results remain sensitive to dataset and methodological variability [40]. Other works use contrast-enhanced modalities (CTP, CTA) to address specific diagnostic tasks such as large vessel occlusion detection [41,42].

However, these approaches are not directly applicable to NCCT-based triage, as they require contrast agents, additional acquisition time, and specialized protocols.

The main findings from the literature are summarized in Table 1.

### 2.4. Research Gaps and Motivation

The reviewed literature reveals three key limitations:

(i) Task formulation gap: existing approaches do not jointly model stroke presence, subtype, and vascular territory, limiting alignment with real-world NCCT triage workflows.
(ii) Pipeline variability: heterogeneous and often underreported preprocessing and training pipelines hinder reproducibility and reliable comparison.
(iii) Limited anatomical modeling: most methods process NCCT as independent slices or selected subsets, discarding anatomical context and underrepresenting challenging regions such as posterior circulation.

These limitations motivate a unified approach that combines: (i) standardized preprocessing, (ii) a clinically aligned multi-class formulation, and (iii) explicit anatomical specialization within a hierarchical framework. The proposed AGTS-Net is designed to address these challenges by integrating these components into a two-stage architecture tailored to NCCT-based stroke triage. In particular, anatomical region pre-classification enables slice-wise classification across the full NCCT study, while improving detection performance in challenging regions such as the posterior fossa.

## 3. Materials and Methods

### 3.1. Architecture Overview

This section describes AGTS-Net, a two-stage cascaded framework designed to classify NCCT images according to stroke type.

In the first stage, a CNN-based pre-classifier (denoted as M*) assigns each NCCT slice to one of five predefined anatomical regions.

In the second stage, a region-specific model performs classification conditioned on the assigned region. The output is a four-class prediction:

- Hemorrhagic stroke (HS);
- Anterior ischemic stroke (AIS);
- Posterior ischemic stroke (PIS);
- No stroke (NS).

**Figure 2:**
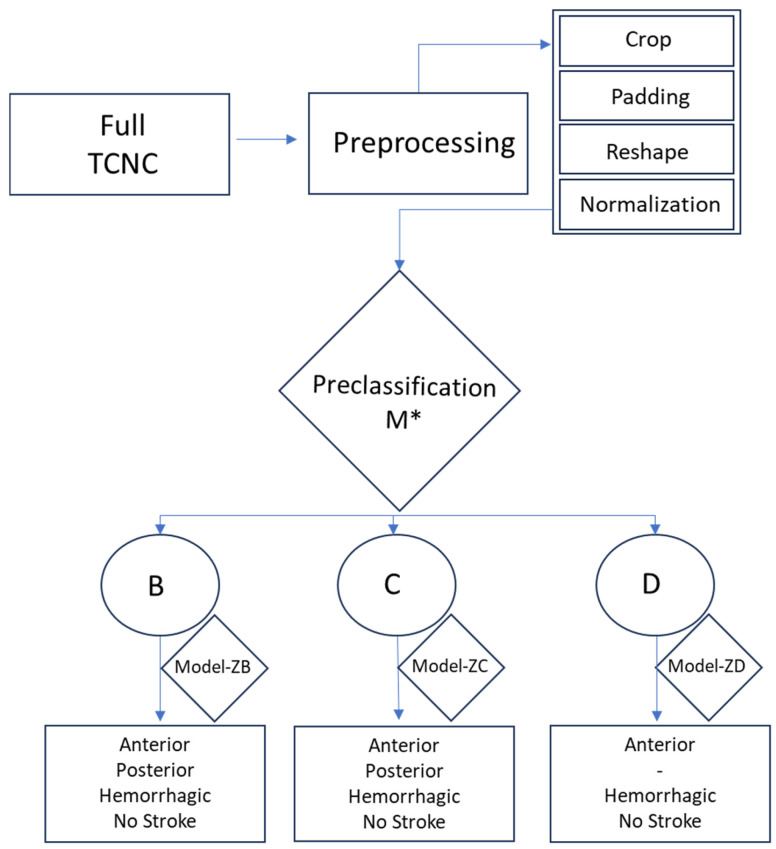
AGTS-Net Architecture.

All slices from a patient’s NCCT study are processed sequentially. Figure 2 illustrates the overall two-stage cascaded architecture.

### 3.2. Image Processing Pipeline

As commonly observed in clinical environments, NCCT images acquired for model training and validation exhibit geometric heterogeneity. Even when generated using the same medical equipment, image size and contrast are not uniform. In addition, the number of slices per patient and slice thickness vary across studies. This variability poses a challenge for deep learning models, which require fixed-size inputs, motivating the formulation of the problem at the slice level.

**Figure 3:**
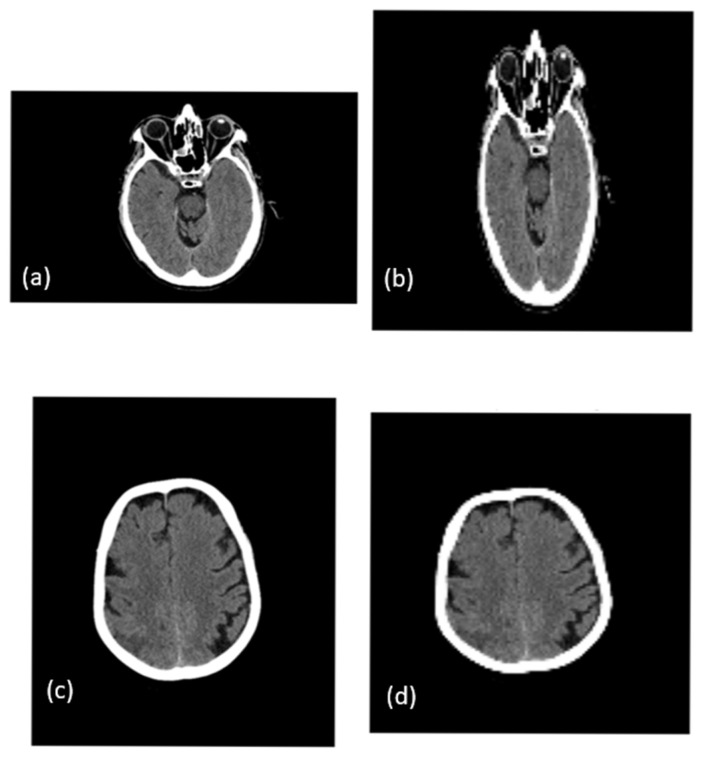
Results of traditional reshape function. (**a**,**c**) raw images. (**b**,**d**) reshaped images.

A common approach in the literature is to apply direct resizing to a target resolution [43,44]. However, this strategy may introduce uncontrolled geometric distortions. Figure 3 illustrates this issue: two images from the same medical centre (a and c) exhibit different spatial resolutions. When a direct reshape operation is applied (b and d), some images become geometrically distorted depending on their original resolution, potentially leading to information loss or erroneous results.

To address this limitation, we propose a standardization algorithm tailored for medical imaging, aimed at improving CNN training by preserving anatomical consistency while ensuring uniform input dimensions.

#### 3.2.1. Image Standardization

A standardization algorithm is developed by exploiting an inherent property of NCCT acquisition: the background outside the skull appears black; i.e., pixel intensity values are zero. Based on this assumption, the following procedure is applied to each image:

- Content detection: Identification of the cranial region by locating the first and last non-zero pixels along both vertical and horizontal axes, defining a bounding rectangle.
- Background removal: All pixels outside the bounding rectangle are removed.
- Squaring and resizing: The resulting image is symmetrically padded with zero-value pixels to form the smallest possible square, which is then resized to 128 × 128.
- Single-channel conversion (after data augmentation): The image is converted to a single-channel (grayscale) representation to reduce model complexity and improve convergence.

**Figure 4:**
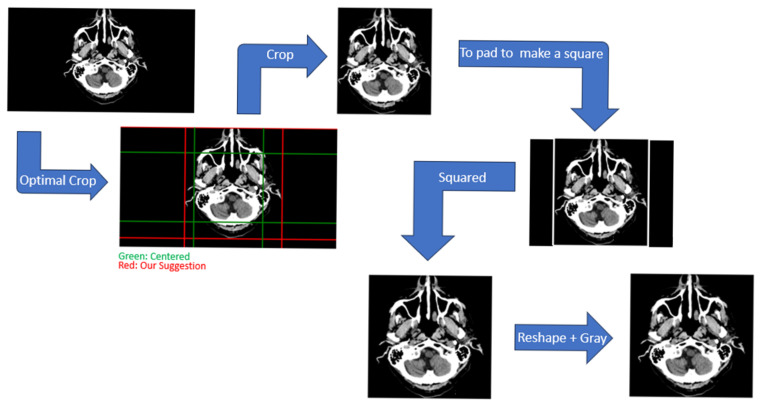
Image standardization process.

The effect of cropping and symmetric padding is illustrated in Figure 4.

#### 3.2.2. Data Augmentation

In deep learning, dataset size and diversity are critical for model generalization [45]. To address the limitations identified in the literature—particularly poor generalization under domain shift and pipeline variability—we apply both geometric and contrast-based data augmentation, introducing controlled variability while preserving anatomical consistency and remaining consistent with clinical acquisition conditions.

The augmentation strategy includes:

- Random rotations within a range of ±45°.
- Horizontal flipping (mirroring) applied to each rotated and original image.
- VOI (Volume of Interest) transformation, applied to each generated sample. This linear intensity transformation enhances pixel values within the range [40, 210], improving intracranial contrast.

Each original image generates 14 augmented variants, increasing the diversity of spatial and intensity distributions and improving robustness to acquisition variability.

To further address pipeline heterogeneity and class imbalance, AGTS-Net performs stratified balancing by anatomical region, limiting the number of samples per class within each region. This prevents majority-class dominance and ensures more stable learning across heterogeneous data distributions.

Moreover, by combining augmentation with region-specific processing, the proposed approach directly addresses the limitation of insufficient anatomical modeling identified in 2.4. In particular, it ensures that underrepresented and structurally complex regions (e.g., posterior circulation) are adequately learned.

Overall, the integration of standardized preprocessing and region-stratified augmentation enables the model to decouple acquisition variability from the diagnostic signal, stabilizing the input distribution and improving robustness under heterogeneous real-world conditions.

### 3.3. CNN-Based Learning Frameworks

AGTS-Net is based on convolutional neural networks (CNNs), whose core operation is convolution. In this process, a filter (kernel) is applied over the input image to extract local patterns such as edges, lines, and textures. Formally, this operation is defined as Equation (1):

(1) $$\left(I\times K\right)\left(i,j\right)=\sum_{m}\sum_{n}I\left(i+m,j+n\right)\cdot K\left(m,n\right)$$

where I denotes the input image, K represents the convolutional kernel, and $\left(i,j\right)$ the coordinates of the resulting activation map. In the proposed architecture, the spatial size of *K* is 3 × 3 in all convolutional layers. The model does not use a single fixed filter matrix; instead, it learns several 3 × 3 filters in each convolutional layer. Specifically, the first convolutional layer uses 32 filters, the second one uses 64 filters, the third one uses 128 filters, and the fourth one uses 256 filters. This operation enables the detection of learned features regardless of their spatial position, providing translation invariance.

Non-linearity is introduced through activation functions, with Rectified Linear Units (*ReLU*) (see Equation (2)) being the most commonly used.

(2) $$ReLU\left(x\right)=max(0,x)$$

*ReLU* promotes sparse activations, mitigates vanishing gradient issues, and accelerates training convergence. To reduce spatial dimensionality and retain the most salient features, max pooling is applied (see Equation (3)),

(3) $$y_{i,j}=\underset{(m,n)\in R_{i,j}}{\max}x_{m,n}$$

where $x_{m,n}$ is the activation value at position (*m*, *n*), $R_{i,j}$ denotes the local pooling region associated with the output position (*i*, *j*), and $y_{i,j}$ is the resulting pooled value. This operation reduces computational complexity while increasing robustness to small spatial variations in the input [46,47].

To prevent overfitting, dropout layers are incorporated, randomly deactivating neurons during training. Additionally, L2 regularization is applied to the network weights to improve generalization on unseen data.

After feature extraction, activation maps are flattened into a one-dimensional vector, which is processed by fully connected layers to perform the final classification. The output layer uses the Softmax function (see Equation (4)) to produce class probabilities.

(4) $$\sigma {\left(z\right)}_{i}=\frac{exp(z_{i})}{\sum_{j}exp(z_{j})}$$

where σ denotes the Softmax function, *z* is the vector of logits produced by the output layer, $z_{i}$ is the logit associated with class *i*, and $\sigma {\left(z\right)}_{i}$ is the predicted probability for class *i*.

Model training is performed using the Adam optimizer, with a tunable learning rate, and categorical cross-entropy as the loss function.

### 3.4. Transfer Learning

In addition to the custom CNN architecture, this work evaluates the integration of AGTS-Net with transfer learning-based classifiers. In the proposed framework, the first stage performs anatomical region preclassification across all NCCT slices. The second stage then applies region-specialized classifiers conditioned on the assigned anatomical zone. Therefore, transfer learning is not considered here as an alternative to the AGTS-Net strategy, but rather as a set of candidate backbone architectures used to implement the second-stage region-specific classification models.

Transfer learning leverages models pretrained on large-scale image datasets and adapts them to a target task by replacing and fine-tuning their final classification layers. This strategy is particularly relevant in medical imaging, where labeled datasets are often limited, heterogeneous, and expensive to annotate [48]. In this study, several representative CNN-based transfer learning architectures were evaluated under the same preprocessing, balancing, training, and evaluation protocol, enabling a fair comparison of their suitability as anatomical zone-specialized classifiers within AGTS-Net.

The evaluated architectures include EfficientNetB0, EfficientNetV2B0, EfficientNetV2B3, ResNet50V2, DenseNet121, DenseNet201, InceptionResNetV2, Xception, and ConvNeXtTiny. EfficientNet and EfficientNetV2 models are based on compound scaling principles, balancing network depth, width, and input resolution to improve accuracy–efficiency trade-offs [49]. ResNet50V2 relies on residual learning, allowing deeper networks to be trained by introducing identity skip connections that mitigate degradation and vanishing gradient problems [50]. DenseNet121 and DenseNet201 use dense connectivity, where each layer receives the feature maps of all preceding layers within the same dense block, promoting feature reuse, improving gradient propagation, and increasing parameter efficiency [51].

InceptionResNetV2 combines inception-style multi-scale feature extraction with residual connections, allowing the model to capture image patterns at different spatial scales while maintaining stable optimization [52]. Xception extends the inception philosophy through depthwise separable convolutions, decoupling spatial and channel-wise feature extraction and reducing computational complexity [53]. Finally, ConvNeXtTiny represents a modern convolutional architecture that incorporates design principles inspired by recent advances in vision models while preserving the inductive biases of CNNs, making it a competitive backbone for image classification tasks [54].

By evaluating these architectures within the same two-stage anatomical framework, the study assesses whether the performance gains are attributable only to a specific CNN design or, more broadly, to the proposed anatomical specialization strategy. This comparison is particularly relevant because each backbone provides a different mechanism for feature extraction, including residual learning, dense feature reuse, multi-scale processing, depthwise separable convolution, compound scaling, and modernized convolutional design. Consequently, the transfer learning experiments provide a robust benchmark for determining the added value of AGTS-Net as a generalizable cascaded framework for NCCT-based stroke classification.

### 3.5. Anatomical Pre-Classifier (M*)

The first stage of AGTS-Net consists of assigning each NCCT slice to a predefined anatomical brain region, providing structured input for the subsequent diagnostic stage.

#### 3.5.1. Definition of Anatomical Regions

Five anatomical regions are defined based on consistent morphological patterns observed in cranial CT slices [55,56]. This partition serves two main purposes:

(i) Reducing structural variability within each region;
(ii) Enabling region-specific specialization, as not all diagnostic classes are plausible at every anatomical level.

**Figure 5:**
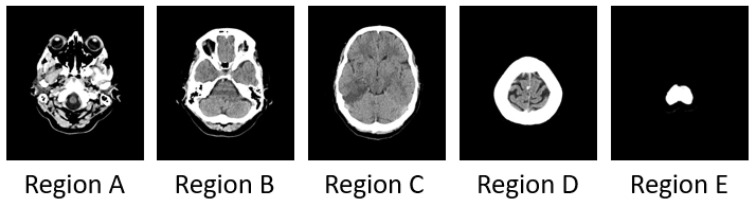
Representative images of each anatomical region.

**Figure 6:**
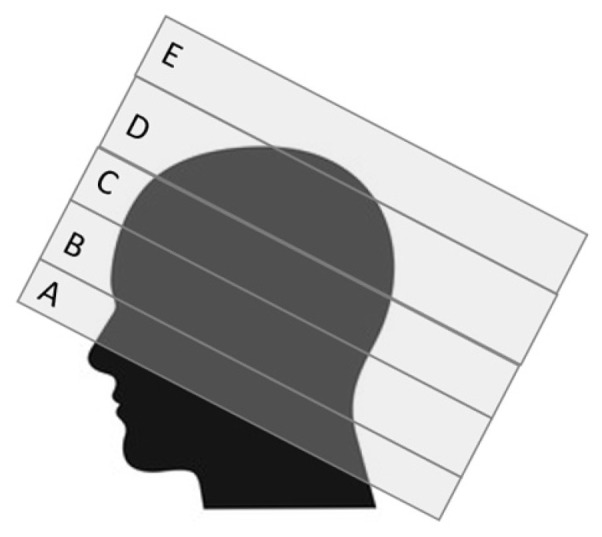
Anatomical region localization.

The defined regions are illustrated in Figure 5 and Figure 6 and are described as follows:

- Region A: Inferior slices near the skull base, from the start of the scan to predominant cerebellar visualization. These slices contain limited brain parenchyma and are therefore excluded from the diagnostic stage.
- Region B: Region extending from cerebellar visualization to the disappearance of sphenoid bones. The presence of these bone structures is a key distinguishing feature and defines the transition to superior regions.
- Region C: Slices where sphenoid bones are no longer visible and ventricular structures appear, characterized by hypodense regions with a high concentration of dark pixels.
- Region D: Superior slices where ventricular structures disappear and cortical sulci predominate, with reduced structural variability.
- Region E: Final slices dominated by bone structures with minimal relevant brain information, which are excluded from classification.

This anatomical partition enables the identification of subsets of slices with homogeneous structural characteristics, improving learning stability and facilitating region-aware classification (see Figure 5 and Figure 6).

#### 3.5.2. Pre-Classifier Architecture (M*)

The anatomical pre-classifier, denoted as M*, is implemented as a lightweight sequential CNN designed to assign each NCCT slice to one of five anatomical regions. The network consists of four convolutional blocks with 3 × 3 kernels and ReLU activation functions. Each convolutional block is followed by a 2 × 2 max-pooling operation and a dropout layer with a rate of 0.30 to reduce overfitting. The number of filters increases progressively across the convolutional stages, from 32 to 64, 128 and 256 filters, allowing the model to learn increasingly complex anatomical patterns.

After feature extraction, the resulting feature maps are flattened and passed to a fully connected layer with 256 neurons and ReLU activation. L2 regularization is applied to this dense layer to further improve generalization, followed by an additional dropout layer with a rate of 0.50. Finally, a Softmax output layer with five neurons provides the probability distribution over the five anatomical regions. The model is trained using the Adam optimizer with a learning rate of 0.0005 and categorical cross-entropy as the loss function.

### 3.6. Region-Specific Model

The second stage of the proposed framework is conceived as a region-specific classification module. After the anatomical pre-classifier (M*) assigns each NCCT slice to a predefined anatomical region, the diagnostic task is delegated to a classifier specialized for that region. This design is motivated by the fact that NCCT slices acquired at different anatomical levels exhibit substantially different structural patterns, tissue distributions, and background characteristics. Therefore, training a single diagnostic model on all slices may increase intra-class variability and make the decision boundaries unnecessarily complex.

**Figure 7:**
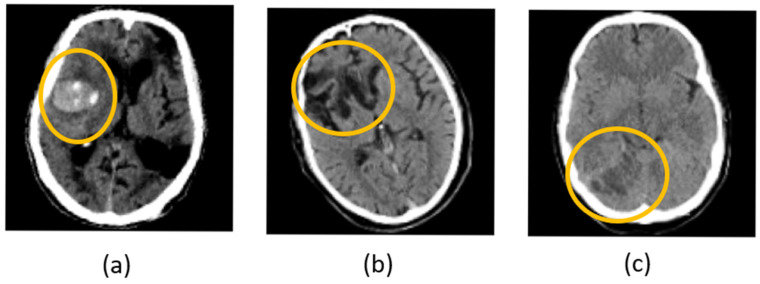
Examples of (**a**) hemorrhagic stroke, (**b**) anterior ischemia, and (**c**) posterior ischemia (highlighted in orange).

By contrast, region-specific models operate on anatomically more homogeneous subsets of images. This allows each classifier to focus on local imaging patterns that are relevant within a given anatomical context, reducing the effect of abrupt structural changes between distant slice levels. In the context of stroke classification, this is particularly important because ischemic lesions may appear as subtle hypodense regions, whereas hemorrhagic lesions usually present as hyperdense areas (see Figure 7). The visibility and discriminative value of these patterns may vary depending on the anatomical region of the scan.

In this work, two complementary strategies are considered for implementing the second-stage classifiers. First, a complete cascaded version of AGTS-Net is developed using custom CNN-based regional classifiers. This configuration allows the proposed two-stage methodology to be evaluated as a full end-to-end diagnostic cascade. Second, several transfer learning architectures are evaluated as alternative region-specific classifiers, including EfficientNetB0, EfficientNetV2B0, EfficientNetV2B3, ResNet50V2, DenseNet121, DenseNet201, InceptionResNetV2, Xception, and ConvNeXtTiny.

The transfer learning experiments are designed to assess whether anatomical specialization improves classification performance beyond the choice of a particular architecture. For this purpose, the pretrained models are evaluated under two settings: training with all anatomical regions jointly and training only with the images belonging to each specific region. This comparison enables the analysis of whether performance gains arise from the backbone itself or from the proposed anatomical partitioning strategy. Therefore, transfer learning is not treated as an alternative to AGTS-Net, but as an additional way of testing the generality and robustness of the region-specialized learning principle.

### 3.7. Model Assembly: AGTS-Net

The overall structure of AGTS-Net is defined as a hierarchical two-stage cascaded framework. Each NCCT slice is first processed through the preprocessing pipeline, where the image is cropped to obtain a standardized spatial representation and pixel intensities are normalized. Once the slice satisfies the required input conditions, it is passed to the anatomical pre-classifier (M*), which assigns it to one of five anatomical regions.

The output of M* determines the subsequent diagnostic pathway. Rather than applying a single classifier to all slices, AGTS-Net routes each image to a region-specific diagnostic model. In the custom CNN implementation of the cascade, these models are denoted as CNN-ZB, CNN-ZC, and CNN-ZD. The routing strategy is defined as follows:

- Region A: No diagnostic prediction is generated, since this region contains insufficient informative brain tissue or is dominated by non-informative structures.
- Region B: The slice is processed by the region-specific classifier CNN-ZB, which produces a prediction within a reduced label space: anterior ischemic stroke, posterior ischemic stroke, or no stroke. Although hemorrhagic stroke may also occur in this anatomical region, the number of representative cases available in the dataset was insufficient to support reliable model training. Therefore, CNN-ZB should not be interpreted as a hemorrhage-exclusion model for this region.
- Region C: The slice is processed by CNN-ZC, which operates over the full diagnostic label space: hemorrhagic stroke, anterior ischemic stroke, posterior ischemic stroke, or no stroke.
- Region D: The slice is processed by CNN-ZD, which produces a prediction within a region-constrained label space: hemorrhagic stroke, anterior ischemic stroke, or no stroke. Posterior ischemic stroke is excluded because structures supplied by the posterior circulation are not represented in this anatomical region.
- Region E: No diagnostic prediction is generated, as this region only contains bony structures and lacks brain parenchyma, making it non-informative for the diagnostic classification stage.

This cascaded decision process ensures that each prediction is conditioned on anatomical context. As a result, the diagnostic output is constrained to clinically and anatomically plausible categories for each slice level. This reduces unnecessary inter-class competition, limits predictions in regions where certain classes are not expected, and allows the classifiers to specialize in the visual patterns most relevant to their corresponding anatomical region.

AGTS-Net combines anatomical preclassification with region-specialized diagnostic learning, producing a structured and clinically constrained framework for NCCT-based stroke classification.

## 4. Experimental Section

### 4.1. Dataset

In this study, two datasets were used:

(i) A proprietary dataset for model training and internal validation;
(ii) A public dataset for benchmarking and external validation.

#### 4.1.1. CAULE Dataset

A proprietary dataset from the hospital Complejo Asistencial Universitario de León (Spain) was used, consisting of 3440 slices from complete NCCT scans of 99 patients. Unlike currently available datasets, which typically provide isolated slices, this dataset includes complete scans per patient, preserving anatomical consistency across slices.

All images were manually annotated by a team of stroke-expert neurologists into the four clinically relevant categories: hemorrhagic stroke (HS); anterior ischemic stroke (AIS); posterior ischemic stroke (PIS) and no stroke (NS) (see Table 2). This labeling scheme provides a more detailed stratification than typical public datasets, which often limit classification to ischemia, hemorrhage, and normal cases.

Furthermore, the dataset includes both patients with confirmed stroke diagnosis and studies acquired for non-cerebrovascular indications. This introduces greater clinical heterogeneity, allowing the evaluation of the model’s ability to discriminate between pathological and non-pathological conditions.

For experimental evaluation, original images were first divided into 80% training/validation and 20% held-out test using stratified sampling. Subsequently, 20% of the training/validation subset was assigned to the validation set, again using stratified sampling. Therefore, before data augmentation, the effective allocation was approximately 64% final training, 16% validation, and 20% testing. This split was performed at the image level rather than at the patient or study level. The held-out test set was not used during model training, model selection, hyperparameter tuning, or early stopping; these procedures were performed exclusively using the training and validation subsets.

Due to ethical restrictions approved by the Complejo Asistencial Universitario de León Ethics Committee, this dataset is not publicly available yet. Data usage is strictly limited to research purposes, ensuring patient privacy.

Additionally, anatomical region labels were manually assigned, as described in Section 3.5.1 (see Table 3).

#### 4.1.2. Kaggle Dataset

To externally evaluate the robustness of the proposed architecture, a publicly available dataset was used, obtained from Kaggle, a widely recognized platform for data science and machine learning competitions [57].

The Kaggle dataset consists of images in both DICOM and PNG formats. It is employed for cross-domain validation; however, it presents a more limited clinical taxonomy, with annotations aggregated into only three categories: hemorrhage, ischemia, and no stroke. This restriction limits its applicability for evaluating finer-grained etiological subtypes.

The distribution of images is shown in Table 4.

### 4.2. Implementation Details

Experiments were conducted using Python 3.12.12 within the Google Colab notebook environment. Data processing and manipulation were performed using NumPy 2.0.2 and Pandas 2.2.2, while model development, training, and inference were implemented using TensorFlow 2.19.0 and Keras. Dataset splitting, performance evaluation, and metric computation were carried out using Scikit-learn 1.6.1. Visualization of training curves, confusion matrices, and performance summaries was performed using Matplotlib 3.10.0 and Seaborn 0.13.2.

All experiments were executed with GPU acceleration available in Google Colab, enabling efficient training of both custom CNN models and transfer learning architectures. The experimental pipeline included three main components: the anatomical pre-classifier (M*), the region-specific CNN classifiers used in the complete AGTS-Net cascade, and the transfer learning models used to benchmark the proposed anatomical specialization strategy.

Prior to training, all NCCT slices were processed using the same preprocessing pipeline. Images were cropped and standardized, converted to grayscale, normalized, and resized to a fixed spatial resolution of 128 × 128 pixels, resulting in input tensors of size 128 × 128 × 1. For compatibility with transfer learning architectures pretrained on natural image datasets, grayscale slices were replicated across three channels when required, yielding input tensors of size 128 × 128 × 3. This ensured that both custom CNNs and pretrained models were evaluated under a consistent preprocessing protocol.

The transfer learning experiments included EfficientNetB0, EfficientNetV2B0, EfficientNetV2B3, ResNet50V2, DenseNet121, DenseNet201, InceptionResNetV2, Xception, and ConvNeXtTiny. For each architecture, the pretrained convolutional base was used as a feature extractor and adapted to the target classification task by adding task-specific classification layers. The models were evaluated under both global and region-specific training settings, allowing comparison between classifiers trained on all anatomical regions and classifiers trained only on slices from a given region.

To reduce class imbalance, the same balancing strategy was applied across the experimental pipeline, combining oversampling and undersampling according to the configuration described in the dataset preparation procedure.

### 4.3. Evaluation Metrics

To evaluate the performance of the classification model, several metrics were used to assess both overall accuracy and class-wise discrimination capability.

Accuracy measures the proportion of correctly classified instances over the total number of predictions, providing a global view of model performance.

Recall quantifies the proportion of true positives correctly identified out of all actual positive cases. This metric is particularly important in medical applications, as it reflects the model’s ability to detect affected cases without missing positive instances.

Precision measures the proportion of predicted positive instances that are actually positive, reflecting the reliability of positive predictions.

To provide a balanced measure that accounts for both false positives and false negatives, the F1-score is used. This metric is defined as the harmonic mean of precision and recall.

These metrics are defined as follows:

(5) $$Accuracy=\left(TP+TN\right)/(TP+TN+FP+FN)$$

(6) Recall=TP/(TP+FN)

(7) Precision=TP/(TP+FP)

(8) F1-Score=2(Precision·Recall)/(Precision+Recall)

where:

- *TP*: true positives;
- *TN*: true negatives;
- *FP*: false positives;
- *FN*: false negatives.

### 4.4. Comparison Benchmarks

To assess the performance of the proposed methodology, several internal comparison benchmarks were defined using the CAULE dataset. First, the complete AGTS-Net cascade based on custom CNN models was evaluated to analyse the effectiveness of the two-stage formulation, including anatomical preclassification and region-specific diagnostic classification. This benchmark represents the full implementation of the proposed framework.

Second, transfer learning architectures were evaluated as alternative diagnostic classifiers within the second stage of the framework. For this purpose, several pretrained models were trained and compared under two different settings: using all anatomical regions jointly and using only the images belonging to each specific anatomical region. This comparison was designed to assess whether anatomical specialization improves classification performance independently of the selected model architecture. Therefore, the benchmark includes both global and region-specialized classifiers based on EfficientNet, EfficientNetV2, ResNet50V2, DenseNet, InceptionResNetV2, Xception, and ConvNeXtTiny architectures.

In addition, an external validation experiment was conducted using the Kaggle dataset exclusively to assess the cross-domain robustness of the anatomical pre-classifier M*. This dataset was not used to evaluate the second-stage diagnostic classifiers, since it does not provide the complete territorial label space required by the proposed diagnostic formulation.

## 5. Results and Discussion

### 5.1. AGTS-Net: Internal Evaluation

The internal evaluation of AGTS-Net was conducted at two complementary levels. First, the anatomical pre-classifier (M*) was evaluated to assess the reliability of the slice-routing stage. Second, the region-specific CNN classifiers were analysed to determine the diagnostic performance of the complete cascaded system once each slice had been assigned to its corresponding anatomical region.

**Figure 8:**
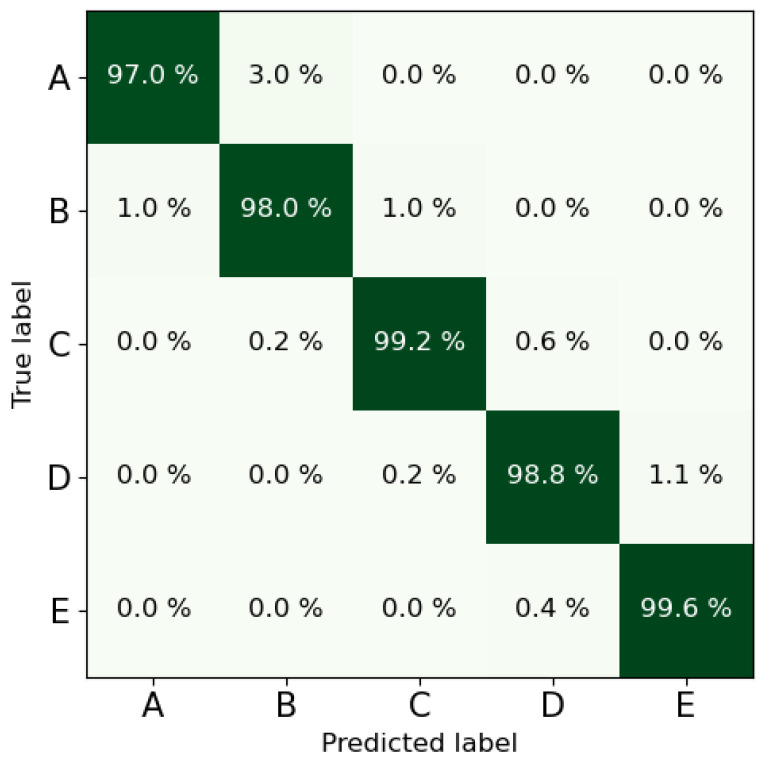
Confusion Matrix of M*.

The anatomical pre-classifier (M*) achieved highly robust performance in the internal validation set, with an overall accuracy of 0.99. As shown in Table 5, precision, recall, and F1-score values ranged between 0.97 and 1.00 across all anatomical regions. These results indicate that the first stage of AGTS-Net provides a reliable anatomical partition of the NCCT study, which is essential for the subsequent region-specific diagnostic classification. The confusion matrix of (M*) (see Figure 8) shows a strong concentration of predictions along the main diagonal, with only a small number of errors between adjacent regions, mainly A–B and C–D. This error pattern is anatomically consistent with the progressive continuity of axial NCCT slices, where transitions between neighbouring anatomical levels may be visually subtle.

Once the anatomical routing stage was validated, the diagnostic performance of the region-specific CNN classifiers was evaluated. Table 6 summarizes the class-wise results obtained by CNN-ZB, CNN-ZC, and CNN-ZD. Overall, the three specialized classifiers achieved high and balanced performance across their corresponding label spaces. This supports the hypothesis that restricting each diagnostic model to anatomically homogeneous slices reduces task complexity and facilitates the learning of region-dependent imaging patterns.

**Figure 9:**
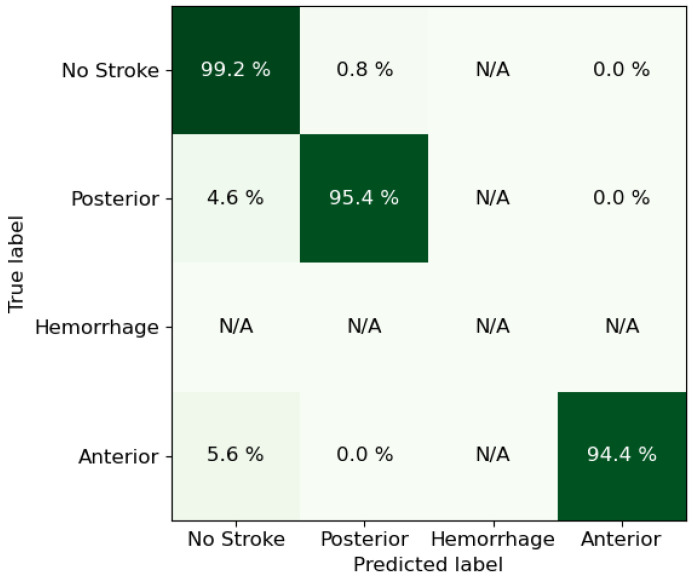
Confusion matrix of CNN-ZB (N/A refers to not applicable).

In Region B, CNN-ZB obtained F1-score values of 0.97 for all diagnostic classes included in this region. The model showed very high precision for posterior ischemic stroke and anterior ischemic stroke, with values of 0.99 and 1.00, respectively. Recall was also high across classes, with the lowest value corresponding to anterior ischemic stroke at 0.94. These results indicate that CNN-ZB is able to discriminate between no stroke, anterior ischemia, and posterior ischemia despite the reduced anatomical level and the absence of hemorrhagic cases in this region. The corresponding confusion matrix (Figure 9) shows limited inter-class confusion, supporting the stability of the classifier in this region.

**Figure 10:**
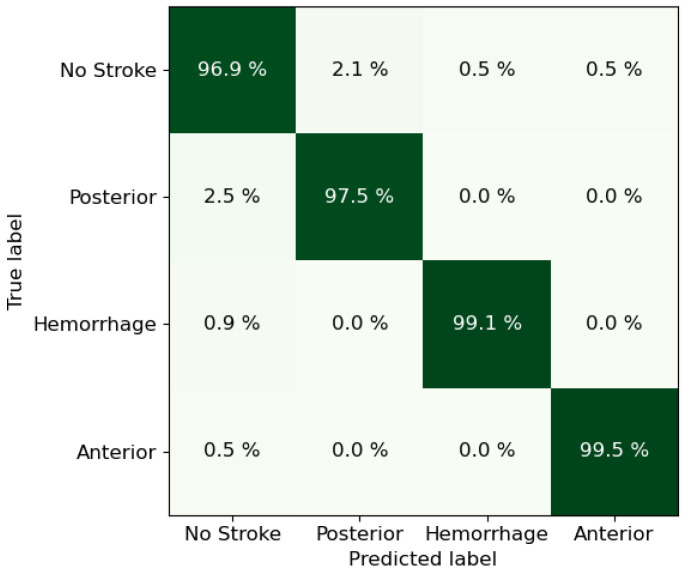
Confusion matrix of CNN-ZC.

Region C represents the most complete diagnostic scenario, since all four diagnostic categories are considered. CNN-ZC achieved the most homogeneous performance among the region-specific classifiers, with precision, recall, and F1-score values ranging between 0.97 and 0.99 for all classes. In particular, hemorrhagic stroke and anterior ischemic stroke reached F1-scores of 0.99, while posterior ischemic stroke and no stroke achieved F1-scores of 0.98 and 0.97, respectively. This indicates that Region C provides a highly informative anatomical context for distinguishing between ischemic, hemorrhagic, and non-stroke patterns. The normalized confusion matrix (Figure 10) further confirms that most predictions are concentrated along the diagonal, with only minor residual misclassifications.

**Figure 11:**
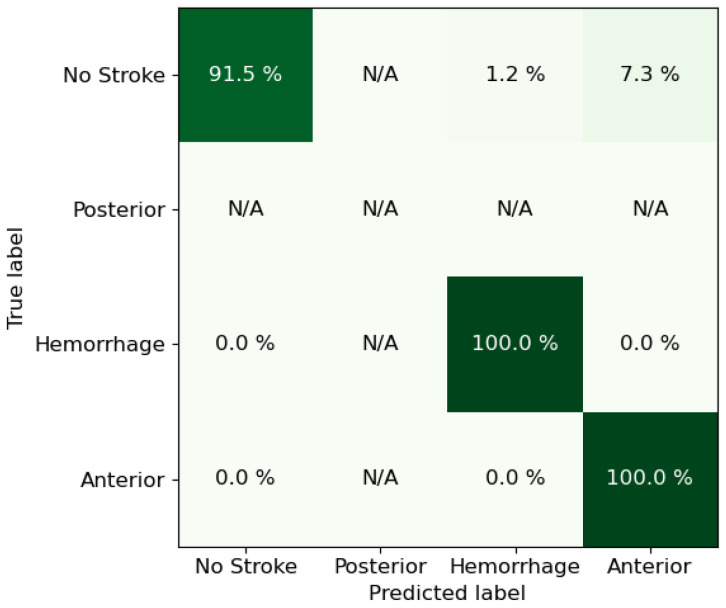
Confusion matrix of CNN-ZD (N/A refers to not applicable).

In Region D, CNN-ZD also achieved high diagnostic performance within its region-constrained label space. The model obtained an F1-score of 0.99 for hemorrhagic stroke and 0.96 for both no stroke and anterior ischemic stroke. Precision was particularly high for no stroke and hemorrhagic stroke, reaching 0.99 and 0.98, respectively. The main limitation was observed in the recall of the no-stroke class, which decreased to 0.91, suggesting that some non-stroke slices may be classified as pathological in this anatomical region. Nevertheless, the high recall obtained for anterior ischemic stroke and hemorrhagic stroke indicates that CNN-ZD prioritizes sensitivity for clinically relevant pathological classes. The confusion matrix (Figure 11) supports this interpretation and shows that the region-constrained formulation maintains a stable diagnostic behaviour.

Taken together, these results show that the complete CNN-based implementation of AGTS-Net provides a robust internal diagnostic pipeline. The anatomical pre-classifier (M*) reliably assigns slices to the appropriate anatomical region, while the region-specific CNN classifiers maintain high class-wise performance within their corresponding diagnostic spaces. Importantly, the reduced or adapted label spaces in Regions B and D prevent the models from producing anatomically implausible predictions, while Region C supports the full diagnostic formulation. This confirms the relevance of anatomical conditioning as a core component of AGTS-Net and supports the use of region-aware classification for NCCT-based stroke analysis.

### 5.2. Impact of Anatomical Specialization

To further assess the contribution of anatomical specialization, several transfer learning architectures were evaluated under two training configurations. First, each model was trained using images from all diagnostic regions jointly and subsequently evaluated on each anatomical zone. Second, the same architecture was trained only with images from the corresponding anatomical region. This comparison was designed to determine whether the performance improvements observed in AGTS-Net are attributable only to a specific CNN design or, more generally, to the proposed anatomical partitioning strategy.

Table 7 summarizes the results obtained for all transfer learning models under both global and region-specific training settings. The reported differences correspond to the performance gain obtained by the region-specific model with respect to the global model evaluated on the same anatomical zone. Overall, the results show a consistent benefit of anatomical specialization. Across the evaluated architectures and regions, macro F1-score improved in 26 out of 27 comparisons, with an average increase of 0.118. The improvement was particularly marked in macro precision, with an average gain of 0.158, indicating that region-specific training reduces false-positive predictions and improves class separation within anatomically homogeneous subsets.

The strongest improvements were observed in Regions B and D. In Region B, the average macro F1-score gain was 0.134. For example, ResNet50V2 increased from an F1-macro of 0.820 when trained globally and evaluated on Region B to 0.974 when trained specifically on Region B. Similarly, DenseNet121 improved from 0.803 to 0.976, and Xception from 0.799 to 0.965. These results suggest that restricting the training data to anatomically consistent slices allows the models to learn more specific decision boundaries, particularly when differentiating between stroke and non-stroke patterns in a reduced anatomical context.

Region C showed smaller but still consistent improvements. This region already provides a relatively informative anatomical context and supports the full diagnostic label space, which may explain why the global models perform comparatively well. Nevertheless, region-specific training improved macro F1-score in all evaluated architectures. For instance, EfficientNetV2B3 increased from 0.822 to 0.940, EfficientNetV2B0 from 0.879 to 0.958, DenseNet201 from 0.907 to 0.959, and Xception from 0.859 to 0.932. The average macro F1-score gain in this region was 0.063, confirming that even in the most complete diagnostic region, anatomical specialization provides additional performance benefits.

The effect was especially relevant in Region D, where the global models often showed high accuracy but lower macro-level performance, indicating class imbalance effects and uneven behaviour across diagnostic categories. Region-specific training substantially improved macro F1-score in most architectures, with an average gain of 0.157. For example, ResNet50V2 improved from 0.687 to 0.950, Xception from 0.669 to 0.958, ConvNeXtTiny from 0.706 to 0.962, and DenseNet121 from 0.722 to 0.937. These results are particularly important because Region D represents an anatomically constrained scenario in which certain diagnostic categories are not plausible. Training models only on this region appears to reduce inter-class confusion and produces more balanced classification behaviour.

Among the region-specific models, DenseNet201 achieved the best macro F1-score in all three anatomical regions, reaching 0.987 in Region B, 0.959 in Region C, and 0.967 in Region D. ConvNeXtTiny also showed highly competitive performance, particularly in Region B, where it achieved an F1-macro of 0.986, and in Region D, where it reached 0.962. EfficientNetV2B0 and EfficientNetV2B3 also benefited from the region-specific strategy, especially in Regions B and C. These results indicate that the proposed anatomical specialization strategy is not architecture-dependent, since improvements were observed across dense connectivity models, residual networks, efficient scaling-based models, depthwise separable convolution architectures, and modern convolutional designs.

### 5.3. Cross-Domain Evaluation of M*

To assess the robustness of the anatomical routing stage under domain shift, the pre-classifier (M*) was evaluated on the external Kaggle dataset.

Compared with the internal validation results, (M*) showed a moderate performance degradation on the external dataset, achieving an accuracy of 0.90 and a macro F1-score of 0.70. This decrease is expected in a cross-domain scenario, where differences in acquisition protocols, image resolution, reconstruction parameters, contrast distribution, and dataset composition may alter the visual characteristics of the input images. Nevertheless, the model preserved a relevant level of anatomical discrimination, particularly in the central regions of the brain, which are the most informative for the subsequent diagnostic stages of AGTS-Net.

As shown in Table 8, Regions B, C, and D maintained relatively stable performance, with F1-scores ranging from 0.89 to 0.93. Region C achieved the highest F1-score, 0.93, followed by Region D, 0.90, and Region B, 0.89. These results indicate that M* is able to preserve reliable anatomical routing in the regions that contain most of the diagnostically relevant brain tissue. Region A showed greater sensitivity to the domain shift, with a precision of 0.65 and an F1-score of 0.77, although its recall remained high at 0.93. This suggests that the model tends to over-assign some external slices to Region A, probably due to differences in slice coverage or acquisition characteristics at the lower anatomical levels. Region E did not show meaningful performance, which is explained by its very limited representation in the external dataset rather than by a systematic failure of the model.

**Figure 12:**
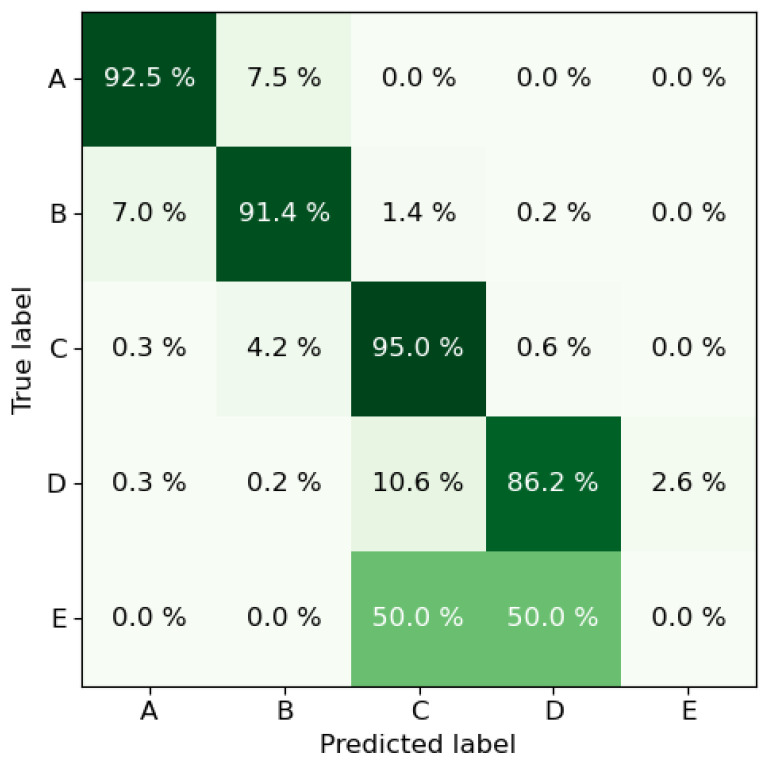
Confusion matrix of M* in the external dataset.

The confusion matrix obtained on the external dataset (Figure 12) shows a pattern consistent with the internal validation behaviour, with most predictions concentrated along the main diagonal and errors occurring mainly between neighbouring anatomical regions. This is coherent with the continuous nature of cranial anatomy in axial NCCT slices, where transitions between adjacent regions are gradual rather than abrupt, so part of the misclassification can be interpreted as boundary uncertainty rather than arbitrary routing errors. From an operational perspective, such errors are less critical than those involving anatomically distant regions, since neighbouring zones often share overlapping visual characteristics and, in some cases, similar diagnostic constraints. Consequently, a limited number of boundary-level routing errors would not invalidate the anatomical guidance provided by M*, particularly given the stable recognition of central regions B, C, and D. Overall, these findings support the use of M* as a robust anatomical guidance module under domain shift and provide preliminary evidence that the first stage of AGTS-Net can generalize beyond the acquisition conditions of the internal dataset.

### 5.4. Practical Implementation

In a potential clinical workflow, AGTS-Net would be designed to process the complete NCCT study automatically, without requiring manual slice selection. After image acquisition, all slices from the NCCT series would be passed through the preprocessing pipeline and evaluated sequentially by the AGTS-Net cascade. For each slice, the anatomical pre-classifier M* would first assign the image to one of the predefined anatomical regions. When the slice belongs to a diagnostically relevant region, the corresponding region-specific classifier would then provide the predicted diagnostic class together with its associated probability.

**Figure 13:**
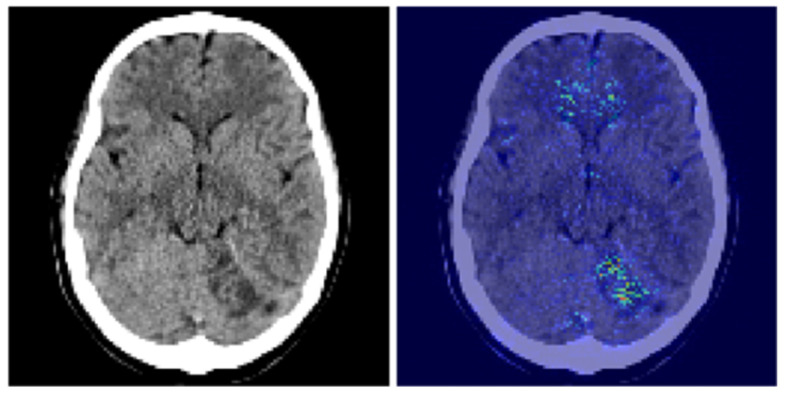
Grad-Cam pixel activation.

To complement the quantitative prediction, explainable artificial intelligence (XAI) analysis was incorporated into AGTS-Net using Gradient-weighted Class Activation Mapping (Grad-CAM) implemented in TensorFlow 2.20.0 (Keras). Grad-CAM uses the gradients of the predicted class with respect to the activations of the last convolutional layers to generate a heatmap that highlights the image regions contributing most strongly to the model decision. In this context, the system provides, for each NCCT slice, a Grad-CAM overlay on the original image, together with the probability associated with each class (Figure 13).

This visual output would enable the clinical user to inspect both the most likely predicted class and the anatomical regions that contributed to the activation [58]. In practice, each original NCCT slice could be displayed alongside its predicted label, class probability, and corresponding Grad-CAM heatmap, allowing radiologists or neurologists to identify slices requiring closer review and to verify whether the model is focusing on anatomically plausible regions.

Importantly, the model output is defined at slice level rather than patient level. Therefore, AGTS-Net should be interpreted as a clinical decision-support and diagnostic guidance tool, not as a system intended to replace medical judgement or to provide a definitive diagnosis. Grad-CAM maps should also not be interpreted as lesion segmentation, but rather as an explainability mechanism to support visual assessment and workflow prioritization during urgent stroke evaluation.

### 5.5. Overall Discussion

The results obtained in this study support the main hypothesis underlying AGTS-Net: incorporating anatomical information before diagnostic classification reduces data heterogeneity and improves the reliability of NCCT-based stroke classification. The proposed framework addresses two key sources of complexity that are commonly present in acute stroke imaging: the subtle appearance of early ischemic changes and the strong anatomical variability across axial slices. By first assigning each slice to an anatomical region and then applying region-specific diagnostic models, AGTS-Net constrains the classification problem to a more homogeneous and clinically plausible decision space.

The internal evaluation of the anatomical pre-classifier (M*) showed very high performance, with an accuracy of 0.99 and class-wise F1-scores between 0.98 and 0.99. This result is particularly relevant because the reliability of the first stage determines the quality of the subsequent routing process. Moreover, the cross-domain evaluation on the external Kaggle dataset showed that M* preserved a relevant level of generalization under domain shift, reaching an accuracy of 0.90. Although the macro F1-score decreased to 0.70, this reduction was mainly influenced by the poor representation of Region E in the external dataset. In contrast, the diagnostically relevant central regions B, C, and D maintained F1-scores between 0.89 and 0.93. These findings suggest that the anatomical routing module remains robust under changes in acquisition conditions, especially in the regions that are most relevant for diagnostic classification.

The region-specific CNN classifiers also achieved strong internal performance. CNN-ZB obtained F1-scores of 0.97 across its diagnostic classes, CNN-ZC reached F1-scores between 0.97 and 0.99 in the full four-class scenario, and CNN-ZD obtained F1-scores between 0.96 and 0.99 within its region-constrained label space. These results are comparable to, and in some aspects extend, previous deep learning studies in stroke imaging. For example, [28] reported an internal accuracy of 0.97 for binary stroke detection, while the AGTS-Net regional classifiers achieved similar or higher values in a more structured diagnostic setting. Likewise, [33] reported accuracies above 0.88 for ischemia–hemorrhage classification in NCCT, whereas the proposed region-specific CNNs reached substantially higher class-wise F1-scores across the evaluated anatomical regions. However, these comparisons should be interpreted with caution, since previous works differ in terms of datasets, label definitions, validation schemes, and imaging protocols.

Compared with studies based on other imaging modalities, such as MRI or DWI, the proposed approach is particularly relevant because it operates on NCCT, which is the most widely used first-line imaging modality in suspected stroke pathways. Previous works have reported high performance for ischemia–hemorrhage classification using MRI-based data, including accuracies above 0.94 [31] and up to 0.98 using CNN-based approaches [32]. Nevertheless, MRI and DWI provide stronger lesion contrast than NCCT and are not always available in emergency settings. Therefore, obtaining high performance from NCCT is clinically meaningful, especially when the model also incorporates vascular territoriality and a “no stroke” category.

The transfer learning experiments further reinforce the value of anatomical specialization. Across the evaluated pretrained architectures, models trained specifically on anatomical regions generally outperformed their global counterparts trained on all regions jointly. This effect was especially evident in macro F1-score and macro precision, indicating that region-specific training improves class balance and reduces false-positive behaviour. Importantly, the improvement was observed across architectures with different design principles, including dense connectivity, residual learning, efficient compound scaling, depthwise separable convolutions, and modern convolutional blocks. This suggests that the gain is not merely architecture-dependent, but is related to the anatomical partitioning strategy itself.

These findings are consistent with the limitations identified in previous literature. Several studies have shown that deep learning models in stroke imaging may achieve high internal performance but suffer from reduced robustness under external validation or changes in acquisition domain. In this context, the cross-domain behaviour of M* is encouraging, since it maintained an external accuracy of 0.90 despite being evaluated on a dataset with different characteristics. This supports the idea that anatomical preclassification may provide a stable intermediate representation for NCCT studies, even when diagnostic labels or acquisition conditions differ across datasets.

## 6. Limitations and Future Work

Although AGTS-Net showed promising results as an anatomically guided framework for NCCT stroke classification, the findings should be interpreted within the scope of the present study: a slice-level decision-support system evaluated on an internal dataset, with external assessment of the anatomical routing stage.

First, the present evaluation was performed at the image level. Therefore, the reported results reflect slice-level performance, which is consistent with the current design of AGTS-Net as a slice-wise decision-support framework. Since the split was not performed at the patient level, intra-patient similarity may have influenced the internal metrics. Accordingly, the high internal performance should not be interpreted as definitive evidence of patient-level generalization. At the same time, the slice-level formulation remains clinically meaningful because stroke studies often contain both affected and non-affected slices within the same patient, and identifying diagnostically relevant slices may support urgent image review. Future studies aimed at clinical deployment should evaluate strict patient-level splitting and aggregation strategies to convert slice-level predictions into patient-level outputs.

Second, external validation of the complete diagnostic cascade was limited by label-space incompatibility in the available public dataset. The Kaggle dataset used in this study provides aggregated categories—hemorrhage, ischemia, and no stroke—but does not distinguish anterior from posterior ischemic stroke. For this reason, external evaluation was restricted to the anatomical pre-classifier M*. These results provide preliminary evidence of cross-domain robustness for anatomical routing, while validation of the full diagnostic stage will require multicentre NCCT datasets with labels aligned with stroke presence, subtype, and vascular territory. Such datasets would not only enable external validation of the complete AGTS-Net cascade, but also increase the patient-level sample size, which is particularly relevant for deep learning and may improve the generalizability of the region-specific diagnostic classifiers.

Third, the anatomical design of AGTS-Net includes methodological choices that should be further evaluated. Anatomical regions were manually assigned using predefined morphological criteria, and future studies should assess labeling reproducibility and sensitivity to boundary slices. In addition, the use of region-specific label spaces reflects anatomical plausibility and reduces unnecessary class competition; however, regional metrics should be interpreted within each anatomical context. Regions A and E were excluded from diagnostic classification because of their limited diagnostic content. Region E contains minimal or no brain parenchyma, whereas Region A, corresponding to the skull-base level, remains challenging due to limited parenchymal content and bone-related artifacts. Region A will therefore be specifically addressed in future developments using additional data and tailored preprocessing. In Region B, hemorrhagic stroke was excluded from the region-specific classifier because the number of representative hemorrhagic cases available at this anatomical level was insufficient for reliable training. Since hemorrhages can occur in Region B, this represents a clinically relevant limitation: in a clinical implementation, additional Region B hemorrhagic cases, a complementary hemorrhage-sensitive screening mechanism, or a conservative fallback rule would be required to reduce the risk of underclassifying hemorrhagic findings.

Finally, AGTS-Net should be considered a decision-support prototype rather than an autonomous diagnostic system. Its potential role in triage would be to process NCCT slices sequentially and highlight relevant images through class probabilities and Grad-CAM explanations, helping clinicians focus attention during urgent stroke assessment. Future work should also compare the proposed 2D anatomical specialization strategy with 2.5D or 3D CNN approaches under harmonized evaluation protocols.

## 7. Conclusions

This study introduced AGTS-Net, a novel anatomically guided two-stage framework for NCCT-based stroke classification. The proposed system was designed to address one of the main limitations of conventional slice-based deep learning pipelines: the loss of anatomical context when all images are processed by a single global classifier. By combining standardized preprocessing, anatomical pre-classification, and region-specific diagnostic models, AGTS-Net constrains the classification problem to more homogeneous and clinically plausible decision spaces.

An important strength of this study is the use of complete NCCT studies from individual patients, rather than isolated or preselected slices. The proprietary CAULE dataset contains 3440 slices from complete NCCT scans of 99 patients, allowing the models to learn from the full anatomical progression of cranial imaging and to capture the variability present across different brain levels. This is particularly relevant for the proposed framework, since anatomical pre-classification and region-specific learning require exposure to the entire structural variability of the brain. By training on complete scans, AGTS-Net reduces the risk of coverage bias and provides a more realistic representation of emergency NCCT interpretation.

The results support the central methodological hypothesis of this work: anatomical regionalization improves classification performance by reducing data heterogeneity and simplifying diagnostic decision boundaries. The anatomical pre-classifier M* achieved highly reliable internal performance, with an accuracy of 0.99 and class-wise F1-scores close to 0.98–0.99. Once this routing stage was established, the region-specific diagnostic classifiers also obtained strong results, with F1-scores between 0.97 and 0.99 in Regions B and C, and between 0.96 and 0.99 in Region D. These findings indicate that conditioning the diagnostic task on anatomical context enables the models to focus on more relevant and region-dependent imaging patterns.

A key contribution of this study is the demonstration that the benefit of anatomical specialization is not limited to the proposed custom CNN implementation. Transfer learning experiments across multiple architectures, including EfficientNet, ResNet, DenseNet, Xception, InceptionResNetV2, and ConvNeXtTiny, showed that region-specific training generally outperformed global training. DenseNet201, for example, improved its macro F1-score from 0.885 to 0.987 in Region B, from 0.907 to 0.959 in Region C, and from 0.941 to 0.967 in Region D. These results suggest that the performance gain is primarily driven by the anatomical decomposition strategy rather than by a particular network backbone.

From a clinical perspective, AGTS-Net provides a structured and actionable output aligned with emergency stroke workflows. Unlike binary approaches that only distinguish stroke from no stroke, or models that assume the presence of stroke, AGTS-Net jointly considers stroke presence, etiological subtype, and vascular territory. This formulation is particularly relevant in urgent NCCT interpretation, where fast differentiation between hemorrhage, anterior ischemia, posterior ischemia, and non-stroke findings can support triage and prioritization.

The incorporation of explainable artificial intelligence through Grad-CAM adds an additional layer of interpretability. The system provides not only class probabilities but also visual activation maps that indicate which regions of the image contributed most to the prediction. This functionality is especially important for clinical adoption, as it allows the radiologist to inspect the model’s focus and use the output as a visual guide. Therefore, AGTS-Net should be understood as a decision-support and productivity-enhancement tool, not as a replacement for medical judgement. Its purpose is to automatically direct attention to potentially relevant areas, reduce cognitive burden, and decrease the probability that subtle findings are overlooked.

Although the complete AGTS-Net diagnostic cascade could not be externally evaluated with the available public dataset because compatible territorial diagnostic labels were not provided, the anatomical pre-classifier M* was assessed on the external Kaggle dataset. This evaluation provided preliminary evidence of robustness under domain shift. Although performance decreased compared with internal validation, M* maintained an accuracy of 0.90, and the diagnostically relevant central regions preserved F1-scores between 0.89 and 0.93. This suggests that anatomical pre-classification captures transferable structural patterns and can act as a stable intermediate representation across heterogeneous acquisition conditions. External validation of the complete diagnostic cascade in multicentre datasets with labels aligned with stroke presence, subtype, and vascular territory remains a priority for future work.

Overall, AGTS-Net contributes both a high-performing slice-level NCCT stroke classification model and a transferable methodological framework. The results show that combining complete-scan training, anatomical decomposition, region-specific learning, and explainability can improve predictive performance while producing outputs that are interpretable and clinically actionable. This modeling philosophy may be extended beyond stroke imaging to other neurological conditions and to other anatomical domains where diagnostic performance is limited by structural variability, subtle imaging findings, or heterogeneous acquisition protocols.

## Figures and Tables

**Table 1:** Summary of key findings and limitations in stroke classification literature.

Category	Task	Key Limitation	References
Binary classification	Stroke vs. no stroke	Generalization performance decreases on external datasets	[28,29]
Etiological classification	Ischemia vs. hemorrhage	Assumes stroke presence; lacks “no stroke” class	[31]
Territorial classification	Vascular territory	Mostly based on MRI or DWI (not NCCT)	[34,38,39]
Contrast-based methods	Advanced diagnostics	Not suitable for NCCT triage workflows	[41,42]
General issue	Pipeline & data	High variability; limited reproducibility	[9]

**Table 2:** Image frequencies by diagnosis and region after data augmentation (CAULE dataset).

	Region B	Region C	Region D
Anterior ischemic stroke (AIS)	75	825	330
Posterior ischemic stroke (PIS)	1905	1665	-
Hemorrhagic stroke (HS)	-	1095	360
No stroke (NS)	2000	1700	400

**Table 3:** Image frequencies by region without data augmentation (CAULE dataset).

Region	Frequency
A	352
B	635
C	1172
D	925
E	356

**Table 4:** Image frequencies by anatomical region (Kaggle dataset).

Region	Frequency
A	187
B	1120
C	3623
D	2080
E	2

**Table 5:** Classification report of the anatomical pre-classifier (M*).

Region	Precision	Recall	F1-Score
A	0.98	0.97	0.98
B	0.98	0.98	0.98
C	0.99	0.99	0.99
D	0.99	0.99	0.99
E	0.97	1.00	0.98

**Table 6:** Classification report of the region-specific CNN classifiers in the diagnostic stage of AGTS-Net.

Model	Definition	Class	Precision	Recall	f1-Score
CNN-ZB	Specialized in region B	No Stroke	0.95	0.99	0.97
		Posterior Stroke	0.99	0.95	0.97
		Hemorrhagic Stroke	N/A	N/A	N/A
		Anterior Stroke	1.00	0.94	0.97
CNN-ZC	Specialized in region C	No Stroke	0.97	0.97	0.97
		Posterior Stroke	0.98	0.98	0.98
		Hemorrhagic Stroke	0.99	0.99	0.99
		Anterior Stroke	0.99	0.99	0.99
CNN-ZD	Specialized in region D	No Stroke	0.99	0.91	0.96
		Posterior Stroke	N/A	N/A	N/A
		Hemorrhagic Stroke	0.98	0.99	0.99
		Anterior Stroke	0.93	0.99	0.96

**Table 7:** Transfer Learning Models Classification Report.

Model	Accuracy	Precision Macro	Recall Macro	F1 Macro	Trained in Region	Evaluated in Region
EfficientNetB0	0.73	0.27	0.28	0.27	ALL	B
	0.57	0.38	0.41	0.39	B	B
	0.26	0.27	0.27	0.13	ALL	C
	0.29	0.23	0.29	0.23	C	C
	0.57	0.34	0.41	0.3	ALL	D
	0.63	0.66	0.63	0.61	D	D
EfficientNetV2B0	0.92	0.78	0.98	0.86	ALL	B
	0.98	0.98	0.98	0.98	B	B
	0.89	0.83	0.95	0.88	ALL	C
	0.96	0.96	0.96	0.96	C	C
	0.98	0.89	0.99	0.94	ALL	D
	0.95	0.96	0.95	0.95	D	D
EfficientNetV2B3	0.9	0.75	0.96	0.83	ALL	B
	0.97	0.97	0.98	0.98	B	B
	0.81	0.78	0.92	0.82	ALL	C
	0.94	0.94	0.94	0.94	C	C
	0.96	0.86	0.98	0.91	ALL	D
	0.95	0.96	0.95	0.95	D	D
ResNet50V2	0.93	0.73	0.97	0.82	ALL	B
	0.97	0.97	0.98	0.97	B	B
	0.92	0.88	0.96	0.91	ALL	C
	0.93	0.93	0.93	0.93	C	C
	0.96	0.65	0.74	0.69	ALL	ZD
	0.95	0.95	0.95	0.95	D	D
DenseNet121	0.93	0.72	0.96	0.8	ALL	B
	0.98	0.98	0.98	0.98	B	B
	0.91	0.86	0.96	0.9	ALL	C
	0.95	0.95	0.95	0.95	C	C
	0.98	0.7	0.74	0.72	ALL	D
	0.94	0.94	0.94	0.94	D	D
DenseNet201	0.94	0.82	0.97	0.89	ALL	B
	0.98	0.98	0.99	0.99	B	B
	0.92	0.86	0.97	0.91	ALL	C
	0.96	0.96	0.96	0.96	C	C
	0.98	0.9	0.99	0.94	ALL	D
	0.97	0.97	0.97	0.97	D	D
InceptionResNetV2	0.92	0.79	0.95	0.86	ALL	B
	0.96	0.96	0.96	0.96	B	B
	0.87	0.81	0.95	0.86	ALL	C
	0.91	0.91	0.91	0.91	C	C
	0.98	0.91	0.98	0.95	ALL	D
	0.94	0.94	0.94	0.94	D	D
Xception	0.88	0.74	0.91	0.8	ALL	B
	0.96	0.97	0.96	0.97	B	B
	0.85	0.81	0.94	0.86	ALL	C
	0.93	0.93	0.93	0.93	C	C
	0.95	0.63	0.74	0.67	ALL	D
	0.96	0.96	0.96	0.96	D	D
ConvNeXtTiny	0.94	0.8	0.96	0.87	ALL	B
	0.99	0.99	0.99	0.99	B	B
	0.92	0.86	0.97	0.91	ALL	C
	0.94	0.94	0.94	0.94	C	C
	0.97	0.68	0.74	0.71	ALL	D
	0.96	0.96	0.96	0.96	D	D

**Table 8:** Classification report of M* on the external Kaggle dataset.

Region	Precision	Recall	F1-Score
A	0.65	0.93	0.77
B	0.87	0.91	0.89
C	0.93	0.92	0.93
D	0.94	0.86	0.90
E	0.00	0.00	0.00

## Data Availability

The original dataset analyzed in this study is available in Zenodo at https://doi.org/10.5281/zenodo.21162781 (accessed on 14 July 2026).

## Abbreviations

The following abbreviations are used in this manuscript:

NCCT	Non-Contrast Computed Tomography
AGTS-Net	Anatomically Guided Two-Stage Network
Grad-CAM	Gradient-weighted Class Activation Mapping
AI	Artificial Intelligence
CNN	Convolutional Neural Network
DWI	Diffusion-Weighted Imaging
CTP	Computed Tomography Perfusion
CTA	Computed Tomography Angiography
HS	Hemorrhagic Stroke
AIS	Anterior Ischemic Stroke
PIS	Posterior Ischemic Stroke
NS	No Stroke
ReLU	Rectified Linear Units
CNN-ZB	Convolutional Neural Network trained in region B
CNN-ZC	Convolutional Neural Network trained in region C
CNN-ZD	Convolutional Neural Network trained in region D
CAULE	Complejo Asistencial Universitario de León
DICOM	Digital Imaging and Communications in Medicine
PNG	Portable Network Graphics
TP	True Positives
TN	True Negatives
FP	False Positives
FN	False Negatives
VOI	Volume of Interest
